# Prenatal gyrification pattern affects age at onset in frontotemporal dementia

**DOI:** 10.1093/cercor/bhab457

**Published:** 2022-01-17

**Authors:** Luke Harper, Olof Lindberg, Martina Bocchetta, Emily G Todd, Olof Strandberg, Danielle van Westen, Erik Stomrud, Maria Landqvist Waldö, Lars-Olof Wahlund, Oskar Hansson, Jonathan D Rohrer, Alexander Santillo

**Affiliations:** Department of Clinical Sciences, Clinical Memory Research Unit, Faculty of Medicine, Lund University, Lund 22184, Sweden; Division of Clinical Geriatrics, Karolinska Institute, Stockholm 14183, Sweden; Department of Neurodegenerative Disease, Dementia Research Centre, UCL Queen Square Institute of Neurology, University College London, London WC1N 3BG, UK; Department of Neurodegenerative Disease, Dementia Research Centre, UCL Queen Square Institute of Neurology, University College London, London WC1N 3BG, UK; Department of Clinical Sciences, Clinical Memory Research Unit, Faculty of Medicine, Lund University, Lund 22184, Sweden; Department of Clinical Sciences Lund, Diagnostic Radiology, Faculty of Medicine, Lund University, Lund 21185, Sweden; Image and Function, Skåne University Hospital, Lund 22185, Sweden; Department of Clinical Sciences, Clinical Memory Research Unit, Faculty of Medicine, Lund University, Lund 22184, Sweden; Memory Clinic, Skåne University Hospital, Malmö 20502, Sweden; Department of Clinical Sciences, Clinical Memory Research Unit, Faculty of Medicine, Lund University, Lund 22184, Sweden; Division of Clinical Geriatrics, Karolinska Institute, Stockholm 14183, Sweden; Department of Clinical Sciences, Clinical Memory Research Unit, Faculty of Medicine, Lund University, Lund 22184, Sweden; Memory Clinic, Skåne University Hospital, Malmö 20502, Sweden; Department of Neurodegenerative Disease, Dementia Research Centre, UCL Queen Square Institute of Neurology, University College London, London WC1N 3BG, UK; Department of Clinical Sciences, Clinical Memory Research Unit, Faculty of Medicine, Lund University, Lund 22184, Sweden

**Keywords:** behavioral variant frontotemporal dementia, cingulate, paracingulate, sulcation

## Abstract

The paracingulate sulcus is a tertiary sulcus formed during the third trimester. In healthy individuals paracingulate sulcation is more prevalent in the left hemisphere. The anterior cingulate and paracingulate gyri are focal points of neurodegeneration in behavioral variant frontotemporal dementia (bvFTD). This study aims to determine the prevalence and impact of paracingulate sulcation in bvFTD. Structural magnetic resonance images of individuals with bvFTD (*n* = 105, mean age 66.9 years), Alzheimer’s disease (*n* = 92, 73.3), and healthy controls (*n* = 110, 62.4) were evaluated using standard protocol for hemispheric paracingulate sulcal presence. No difference in left hemisphere paracingulate sulcal frequency was observed between groups; 0.72, 0.79, and 0.70, respectively, in the bvFTD, Alzheimer’s disease, and healthy control groups, (*P* = 0.3). A significant impact of right (but not left) hemispheric paracingulate sulcation on age at disease onset was identified in bvFTD (mean 60.4 years where absent vs. 63.8 where present [*P* = 0.04, Cohen’s *d* = 0.42]). This relationship was not observed in Alzheimer’s disease. These findings demonstrate a relationship between prenatal neuronal development and the expression of a neurodegenerative disease providing a gross morphological example of brain reserve.

## Introduction

Frontotemporal dementia (FTD) is the second most common cause of early-onset dementia (age at onset <65 years). A genetic etiology is observed in approximately 15–30% of cases of FTD (Greaves and Rohrer 2019). In cases where a genetic mutation has not been isolated, aside from the possible exceptions of age and single nucleotide polymorphisms encompassing the trans-membrane protein 106B, there are no established disease risk factors (Nilsson et al. 2014; Ferrari et al. 2019). Behavioral variant frontotemporal dementia (bvFTD) is the most common clinical subtype of FTD, accounting for 60% of all cases (Onyike and Diehl-Schmid 2013). BvFTD is characterized by progressive changes in personality and behavior, which include disinhibition, apathy/inertia, loss of sympathy/empathy, perseverative/compulsive behaviors, hyperorality, and a dysexecutive neuropsychological profile (Rascovsky et al. 2011).

Together with the insula cortex, the anterior cingulate cortex (ACC) is a focal point of neurodegeneration in bvFTD, afflicted early and extensively in the disease process (Seeley 2010). On a physiological level, the ACC anchors the “salience network” which provides a key function in a range of executive cognitive functions including cognitive control, typically affected in bvFTD (Zhou et al. 2010). On a macroscopic level, the anterior cingulate displays a high degree of heterogeneity with extensive individual and hemispheric variability (Palomero-Gallagher et al. 2008; Del Maschio et al. 2019). One stable variable is the presence of the paracingulate gyrus and its respective sulcus, the paracingulate sulcus (PCS). The PCS is a tertiary sulcus which develops, where present in the second to third gestational trimesters and is observed in 30–60% of hemispheres (Chi et al. 1977; Paus et al. 1996; Yücel et al. 2001; Fornito et al. 2004). Neighboring banks of the ACC and paracingulate cortex (PCC) are cytoarchitecturally indistinguishable, more similar to one another than any other cytoarchitectonic area in the medial wall of the cerebrum (Smith 1907; Palomero-Gallagher et al. 2009) as such there is suggestion that the PCC may be regarded as part of the ACC (Palomero-Gallagher et al. 2008).

Paracingulate gyral activation has been observed during performance in a variety of tests of executive, higher-order processing including spatial working memory and planning (Fornito et al. 2004). Consequently, the PCC is suggested to serve a role in cognition, interacting with regions of the lateral prefrontal cortex to mediate performance across a wide variety of cognitively demanding (verbal and nonverbal) tasks, particularly those involving executive cognitive processing (Fornito et al. 2004). Presence of a paracingulate gyrus is determined deductively in morphological studies through identification of PCS. Individuals with leftward hemispheric asymmetry of ACC sulcation (the presence of a PCS in the left hemisphere with absence in the right) exhibit a performance advantage across several higher-order functions that draw on cognitive control, including reality monitoring, inhibitory control, and verbal and spatial working memory (Fornito et al. 2004). In healthy individuals, there is a hemispheric asymmetry of paracingulate sulcation such that paracingulate sulcation is more prevalent in the left hemisphere (Paus et al. 1996; Yücel et al. 2001; Yücel et al. 2002; Le Provost et al. 2003). Conversely, in schizophrenia, a disease with implied pathological dysfunction of the ACC, a reduction in the magnitude of leftward-dominant paracingulate sulcation asymmetry is an established biomarker pointing to a potential neurodevelopmental aberration (Yücel et al. 2002; Le Provost et al. 2003).

To the best of the authors’ knowledge, morphological analysis of paracingulate sulcation is unstudied in the neurodegenerative diseases. The primary aim of this study is to examine whether a reduced prevalence of left hemisphere paracingulate sulcation is present in bvFTD, akin to the case in schizophrenia. Secondary aims of the study are to explore right hemisphere PCS frequency, interhemispheric asymmetry, and the impact of hemispheric sulcation on age at onset (AAO) in bvFTD.

## Materials and Methods

### Participants

A cross-section study was adopted with the study population drawn from the following cohorts recruited from memory clinics at University Hospitals in Sweden and the United Kingdom: LUPROFS (Lund Prospective Frontotemporal Dementia study), BioFINDER-2 (Skåne University Hospital, Sweden [NCT03174938]), the Longitudinal Investigation of FTD study (University College London, UK), and Empathy in FTD (Lund-Stockholm-Umeå, Sweden). Detailed cohort descriptions can be found in the Supplementary Material.

The study population consisted of three groups. The first, a bvFTD group, diagnosed in accordance with revised International bvFTD Consortium criteria (Rascovsky et al. 2011). Following genetic screening, individuals with a known Chromosome 9 open reading frame 72 (*C9orf72*), Progranulin (*GRN*), or Microtubule Associated Protein Tau (*MAPT*) mutation were excluded from study. All but seven of the included individuals were genetically screened. The bvFTD group consisted of 105 participants (2 possible, 93 probable, and 10 definite bvFTD), 70 males and 35 females with a mean age at scan (AAS) of 66.9 years (SD 8.15) and AAO of 62.2 (SD 8.23). The second group consisted of 110 cognitively healthy controls (HCs), 72 males and 38 females with a Mini Mental State Examination (MMSE) score of $\ge$26 and mean AAS 62.4 (SD 12.11). The final group, acting as a neurodegenerative disease comparator, consisted of 92 individuals with Alzheimer’s disease (AD) dementia, of which 62 were male and 30 were female, mean AAS 73.3 (SD 6.54). AD participants were diagnosed according to clinical and biochemical criteria outlined in the Supplementary Material.

Groups were sex-matched in view of an established gender difference in hemispheric paracingulate sulcation described in two studies of healthy individuals (Paus et al. 1996; Yücel et al. 2001). The final study population consisted of 307 participants across three groups detailed in Table 1.

**Table 1:** Study population and results

	bvFTD	AD	HC	Statistical Test
Participants	105	92	110	
Age, years (SD)	66.9 (8.2)	73.3 (6.5)	62.4 (12.1)	ANOVA, *P* < 0.001
Age at onset, years (SD)	62.2 (8.2)			
Sex	M70:F35	M62:F30	M72:F38	Chi-Squared, *P* = 0.96
Education, years^a^ (SD)	11.53 (3.34)	12.18 (4.39)	13.47 (3.83)	ANOVA, *P* = 0.02
Hemispheric ACC sulcation				
Double left	76 (0.72)	73 (0.79)	77 (0.7)	Chi-Squared, *P* = 0.30
Double right	60 (0.57)	56 (0.61)	63 (0.57)	Chi-Squared, *P* = 0.84
Paracingulate sulcal frequency	0.65	0.70	0.64	
Ratio of left PCS: Right PCS	1.27	1.30	1.22	
Sulcal pattern				
Double/double (dd)	42 (0.4)	46 (0.5)	45 (0.41)	
Double/single (ds)	34 (0.32)	27 (0.29)	32 (0.29)	
Single/double (sd)	17 (0.16)	10 (0.11)	18 (0.16)	
Single/single (ss)	12 (0.11)	9 (0.1)	15 (0.14)	
Asymmetry (ds + sd)	51 (0.49)	37 (0.4)	45 (0.41)	
Symmetry (dd + ss)	54 (0.51)	55 (0.6)	60 (0.59)	
Asymmetry index (ds:sd)	2.00	2.70	1.78	
Frontal lobe atrophy^b^	1.36	0.70	0.06	

*Notes*: Demographic and results data for behavioral variant frontotemporal dementia (bvFTD), Alzheimer’s disease (AD), and healthy control (HC) groups. Standard deviation (SD). Sex data: Male (M). Female (F). Paracingulate sulcal frequency: Displayed as the relative frequency of hemispheres with a present PCS to total number of hemispheres. ACC sulcation patterns were ascribed to individuals based on a combination of their left and right hemispheric AC sulcation. As such, “single/single” denotes only ACS presence in both hemispheres, “double/double”, ACS and PCS in both hemispheres, “double/single” (i.e., leftward ACS asymmetry), PCS present in the left hemisphere but absent in the right and “single/double” (i.e., rightward ACS asymmetry), and PCS present in the right hemisphere and absent in the left. Frequency displayed as whole number and relative frequency displayed in brackets. Asymmetry: Frequency of “double/single” + “single/double”, relative frequency displayed in brackets. Symmetry: Frequency of “double/double” + “single/single”, relative frequency displayed in brackets. Asymmetry index: Calculated as a ratio of leftward ACS asymmetry (double/single) to rightward ACS asymmetry (single/double).

a Education, years data available for 62/105 FTD, 88/92 AD, and 64/110 HC individuals.

b Mean of Frontal Lobe Atrophy ratings, analyzed in accordance with Kipps criteria for frontal lobe atrophy (Kipps et al. 2007).

All study participants gave informed consent in accordance with the Declaration of Helsinki prior to inclusion in their native cohorts. Native studies were conducted with the approval of respective local ethics committees; further information on this can be found in the Supplementary Material.

### Magnetic Resonance Image Acquisition and Image Analysis

High-resolution volumetric whole brain T1-weighted magnetic resonance (MR) images were obtained using 3.0 Tesla machines with a minimum spatial resolution of 1.1 × 1.1 × 1.2 mm. Protocol details are provided in the Supplementary Material.

Prior to analysis, images were anonymized and visually inspected for the presence of significant movement artifact or co-morbid intracranial pathology, which may have obscured rating of PCS. Two individuals were removed on this basis and were not accounted for in the study population. One, a HC with an MR scan distorted by movement artifact, and the other, an individual with AD whose PCS was obscured by the presence of a large right frontal meningioma. Images were imported into MANGO (Multi-image Analysis GUI, v 4.0, http://ric.uthscsa.edu/mango/mango.html, The University of Texas Health Science Center) software and prepared, aligning the *x-*axis in the sagittal plane with the bicommissural line (AC–PC). Further *y*- and *z*-axes rotational corrections were performed in order to ensure optimal orientation for analysis.

### Paracingulate Sulcus Measurement and Classification Criteria

Garrison’s established protocol for PCS identification and measurement was refined and utilized for PCS classification (Garrison et al. 2015). A complete description of this protocol is outlined in the Supplementary Material.

The cingulate sulcus (CS) is initially identified 4 mm laterally from the midline (*x* = 0) on T1 sagittal brain MR images. The PCS is then identified as the sulcus running predominantly dorsal and parallel to the CS. The PCS depth must be clearly visible in four or more consecutive sagittal slices (>4 mm). The anterior limit of the PCS is identified as the point at which the sulcus begins to move posteriorly and parallel to the CS from an imaginary line perpendicular to the AC–PC line (Yücel et al. 2001). The PCS is measured from this point using MANGO’s (Multi-image Analysis GUI, v 4.0, http://ric.uthscsa.edu/mango/mango.html, The University of Texas Health Science Center) “Trace Line” function until its end point, the point where the sulcus is interrupted by a distinct predominantly vertical gyrus deemed non-paracingulate in nature. The PCS must originate in the first quadrant (although it may extend beyond the first quadrant) on a sagittal plane where x0, y0 marks the point of the anterior commissure after images have been aligned in the AC–PC plane.

A binary sulcation classification was utilized where-by hemispheric (right and left) PCS presence in each individual was identified as either “present” (≥20 mm) or “absent” (≤19 mm), as is standard among PCS classification protocols (Ono et al. 1990; Yücel et al. 2002; Le Provost et al. 2003; Garrison et al. 2015; Del Maschio et al. 2019). Quantitative measures and sub-classification of the PCC sulcal morphology including sulcal width, depth, or length were not analyzed since these cortical features vary with age (Clark et al. 2010; Cachia et al. 2016).

**Figure 1:**
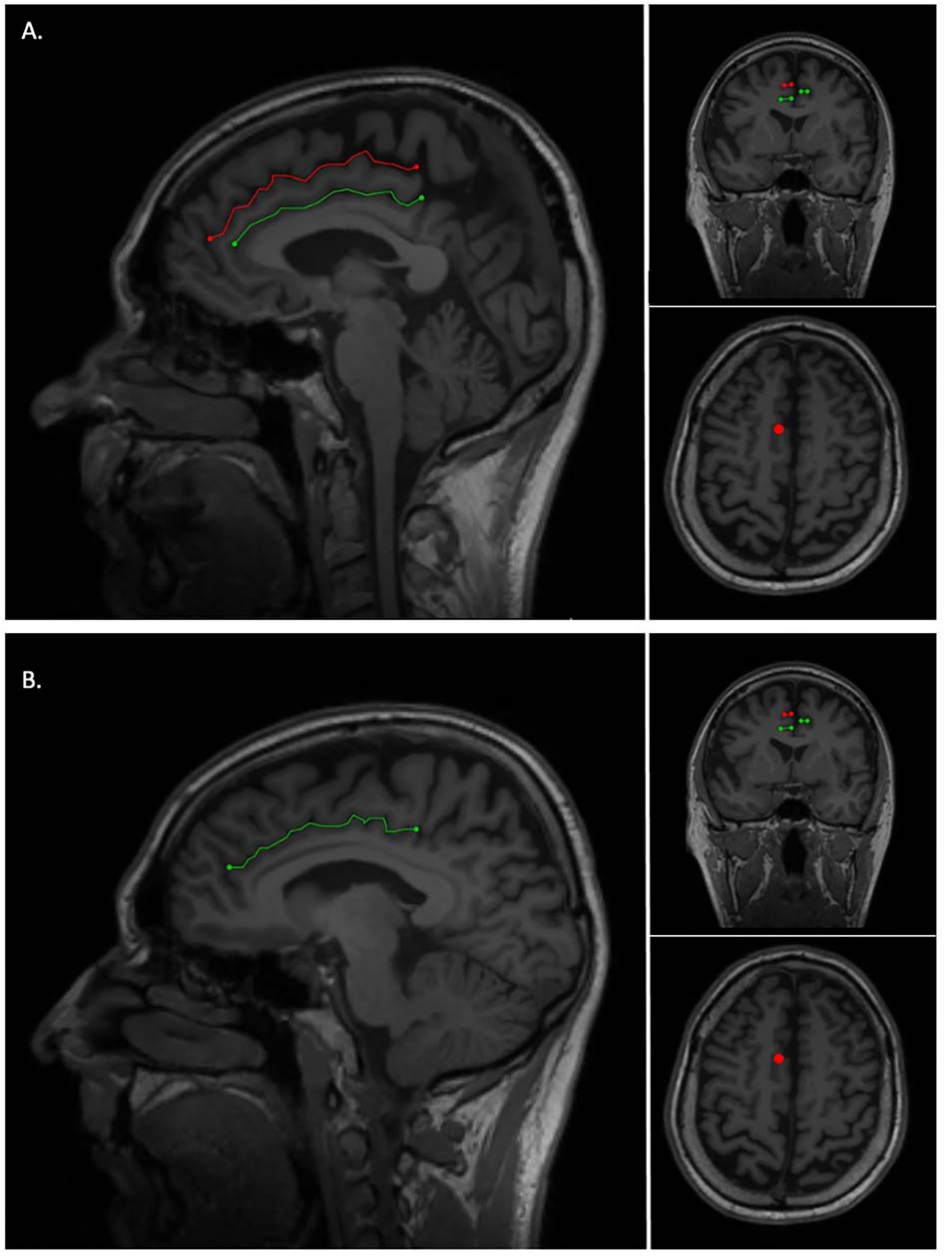
Cingulate and paracingulate sulci identification and measurement. A 62-year-old male with probable bvFTD displays a leftward pattern of paracingulate asymmetry (“double/single”). (*A*) Sagittal slice (left) of the left hemisphere 3 mm left of the midsagittal line displaying a prominent left PCS (red) and CS (green). (*B*) Sagittal slice (left) of the right hemisphere 3 mm right of the midsagittal line displaying an CS (green) with absence of a rightPCS.

Hemispheric anterior cingulate sulcation was termed according to standard nomenclature, such that where a PCS was present, sulcation was classified as “double” (representing the presence of both a CS and a PCS) and where absent classified as “single” (CS only). Figure 1 displays an individual with a “double/single” (Leftward-dominant) pattern where the PCS is present in the left hemisphere but absent in the right. Sulcation ratings were performed by two raters, L.H. and A.S., who were blinded to individuals’ demographic data and study group. Disagreement between raters was resolved by consensus.

Prior to study reliability studies were conducted using the methods described above in an independent cohort of 30 healthy individuals. Intra-rater agreement, L.H. versus L.H., was 98.33%, *Cohens Kappa* 0.96. Inter-rater agreement, A.S. versus L.H., was 94.44%, *Cohens Kappa* 0.85.

Consideration was given to the potential impact of frontal lobe atrophy on PCS ratings. Consequently, frontal lobe atrophy rating was conducted according to a 5-point scale described by Kipps et al. (2007). No significant association between the degree of frontal lobe atrophy and PCS frequency was identified in either the study population (Chi-squared = 6.79, df = 4, *P* = 0.15) or in the bvFTD group when analyzed in isolation (Chi-squared = 5.60, df = 4, *P* = 0.23).

### Statistical Analysis

Statistical analysis was performed using R software (R CoreTeam 2016, https://www.r-project.org/).

Assuming a similar sulcation difference may be found in this study, data from two studies in Schizophrenia (Yücel et al. 2002; Le Provost et al. 2003) were utilized in order to perform a power calculation using Fisher’s exact test. Two hypotheses were accounted for (H1: bvFTD vs. HC, H2: BvFTD vs. AD), as such a Bonferroni correction was performed, “alpha” 0.025 (0.05/2), “power” 0.8. Calculations identified a desired population of between 47 and 96 participants per group.

Differences in hemispheric ACC sulcation frequencies were calculated using chi-squared tests. Intragroup asymmetry differences were calculated using McNamar’s chi-squared test for asymmetry. Independent student’s *t*-tests were performed for analysis of the impact of ACC sulcation on AAO in the bvFTD group. Effect sizes were calculated according to Cohen’s d. Linear regression models were performed in order to evaluate covariate effects of education and sex on study outcomes.

Statistical analyses, procedures related to our primary and secondary aims, and power calculations for the primary aim were a priori pre-registered and may be accessed directly at https://osf.io/h6t2z.

### Data Availability

Anonymized data will be shared by request from a qualified academic investigator for the sole purpose of replicating procedures and results presented in the article if data transfer is in agreement with EU legislation on the general data protection regulation and decisions and by the relevant Ethical Review Boards, which should be regulated in a material transfer agreement.

## Results

Contrary to our primary hypothesis, no difference in left hemisphere paracingulate sulcal frequency was observed between groups, with respective frequencies of 0.72, 0.79, and 0.70 in the bvFTD, AD, and HC group (Chi-squared = 2.38, df = 2, *P* = 0.30). Furthermore, sulcation frequency of the right paracingulate was also similar across groups: bvFTD group 0.57, AD 0.61, and HC 0.57 (Chi-squared = 0.36, df = 2, *P* = 0.84). Results are displayed in Table 1.

Additionally, similar ACC sulcal pattern frequencies were also observed between groups (Chi-squared = 3.53, df = 6, *P* = 0.74). A leftward asymmetry was identified in all groups with the following indices: bvFTD; 2.0 (McNemar’s chi-squared = 5.02, df = 1, *P* = 0.03), AD; 2.7 (McNemar’s chi-squared = 17.28, df = 1, *P* ≤ 0.01), and HC; 1.78 (McNemar’s chi-squared = 3.38, df = 1, *P* = 0.07).

**Figure 2:**
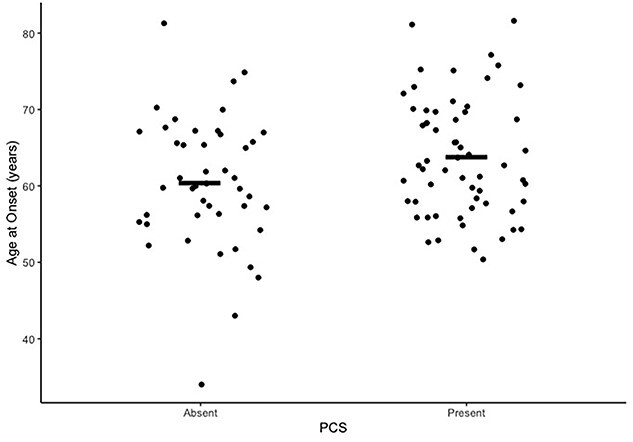
Age at symptom onset by right hemisphere PCS presence in bvFTD. Black dots represent study individuals. Black lines represent group mean age at symptom onset.

In intragroup analysis, a significant impact of right hemispheric paracingulate sulcation on AAO was observed in the bvFTD group. Mean AAO in participants with “single” right ACC sulcation was 60.4 years versus 63.8 in those with “double” right ACC sulcation (*t* = −2.09, df = 88.6, *P* = 0.039, Cohen’s d = 0.42), Figure 2. This was also the case when calculations were repeated using AAS. Mean AAS in those with “single” right ACC sulcation was 64.9 versus 68.5 in those with “double” right ACC sulcation (*t* = −2.26, df = 84.1, *P* = 0.026, Cohen’s d = 0.46). No effect of ACC sulcation on AAO was observed in the left hemisphere, mean age “single” 62.72 versus “double” 62.17 (*t* = 0.34, df = 62.0, *P* = 0.74, Cohen’s d = 0.07).

No impact of paracingulate sulcation on AAS was identified in the AD group in either the left (*t* = −0.53, df = 35.8, *P* = 0.6) or right (*t* = −0.97, df = 75.2, *P* = 0.33) hemisphere. Similarly, no impact on AAS by either left (*t* = 0.45, df = 71.48, *P* = 0.66) or right (*t* = 0.21, df = 92.34, *P* = 0.83) hemisphere paracingulate sulcation was observed in the HC group.

In order to explore the potentially confounding effect of sex on AAO in the bvFTD group, post hoc multiple linear regression analyses were performed adding sex as a covariate to a model with PCS presence as the independent variable. There was no significant impact of sex on AAO in models of either left (*P* = 0.22) or right (*P* = 0.17) PCS frequencies and the effect of right PCS presence on AAO was retained after adjusting for sex (*P* = 0.03). Furthermore, no influence of sex was identified on either right (Chi-squared = 0.95, df = 1, *P* = 0.33) or left (Chi-squared = 1.72, df = 1, *P* = 0.19) PCS presence.

In the FTD group, no difference was observed in years of education between individuals with “single” versus “double” right (*t* = −0.92, df = 48.90, *P* = 0.36) or left (*t* = −1.43, df = 19.38, *P* = 0.17) ACC patterns. Additionally, there was no significant correlation between education and AAO (*r* = −0.16, CI −0.40–0.09, *P* = 0.21). In order to further explore whether education influenced the relationship between PCS and AAO in the bvFTD group, a linear model was created with PCS as an independent variable and education as a covariate for individuals with available education data. Right hemispheric PCS presence was associated with a significantly greater AAO after adjusting for education (*P* = 0.007), in turn education did not significantly impact AAO in this model (*P* = 0.10). Using a similar model, no impact of either left hemisphere PCS presence (*P* = 0.8) or education (*P* = 0.24) was observed on AAO. In addition, no interaction effect of education and either right (*P* = 0.76) or left (*P* = 0.60) PCS presence was seen onAAO.

## Discussion

We described a novel disease-modifying factor in bvFTD, such that increased right ACC sulcation is associated with a delay in disease onset. This finding was not identified in the AD group suggesting that this factor is independent to bvFTD. Contrary to our primary hypothesis and unlike studies performed in schizophrenia (Yücel et al. 2002; Le Provost et al. 2003) which provided the impetus for this study, a reduction in left ACC sulcation was not observed in bvFTD or AD. AC sulcation frequencies were also similar in right hemispheres across study groups indicating that this negative finding is not a type II error and anterior cingulate gyrifications patterns therefore do not seem to be a risk factor for bvFTD. As has been observed in studies of healthy individuals, a leftward asymmetry dominance was present in all groups.

Brain gyrification indices and subsequent sulcation in humans reflect the density of intrinsic neural connectivity, as such there is biological plausibility for the impact of ACC sulcation on disease onset in bvFTD (Welker 1990). The tension-based morphogenesis (TBM) model and its revision, the differential expansion sandwich plus model, consider cortical folding to be partially pathway-specific, dependent on mechanical tensions along axons, dendrites, and glial processes connecting different brain regions (Van Essen 1997, 2020). In turn, right PSC sulcation may indicate marked local connectivity within paralimbic cortices (Brodmann’s area [BA] 32) and adjacent regions (BA 6, 8, and 9; Le Provost et al. 2003), which may provide a degree of resistance to neurodegeneration in bvFTD, delaying disease expression. The same model has been used to explain findings in schizophrenia where it is suggested that this disease may arise as a consequence of weaker cortical connectivity due to the lower frequency of left ACC sulcation and correspondingly reduced gyrification (Le Provost et al. 2003). More specifically, a further possible mechanism whereby cingulate variability may translate into the pathophysiology of bvFTD is through disruption of the salience network, a basic intrinsic resting state network anchored in the anterior cingulate and characteristically disrupted in bvFTD (Zhou et al. 2010).

Together with the insula, the ACC is populated by von Economo neurons (VEN), unique pyramidal neurons selectively targeted in FTD (Santillo et al. 2013). The impact of cingulate variability on VEN density is to our knowledge unknown. Gyrification, however, has been shown to affect the relative distribution of BA24 subregions (BA24a, b, and c) (Vogt et al. 1995), which contain differing VEN densities (Nimchinsky et al. 1995) and may explain how cortical folding variations influence disease expression in bvFTD.

As the PCS arises during gestation (Chi et al. 1977; Del Maschio et al. 2019), our finding is therefore an example of how neurodevelopmental variation may translate into the expression of a neurodegenerative disease. As for other neurodegenerative diseases, individual resilience to the disease process likely plays a role in bvFTD. Currently resilience is operationalized as consisting of brain reserve, brain maintenance, and cognitive reserve (Stern et al. 2020). PCS presence is, using this terminology regarded as a facet of brain reserve, being a congenital “neurobiological capital” (Stern et al. 2020). Individuals with FTD with higher education (Perneczky et al. 2007; Premi et al. 2012, 2020), occupational attainment (Borroni et al. 2009), and degree of occupational engagement in social and cognitive control skills (Dodich et al. 2018) but comparable clinical severity have shown more global and regional functional cerebral impairment across a range of imaging modalities. Although education has not been directly shown to influence risk for or AAO in FTD in these studies, they demonstrate that individuals with a greater cognitive reserve can cope with an increased burden of disease (Premi et al. 2012, 2020). Thus, education is an important covariate in this study; however, it was not found to impact upon AAO in the bvFTD group and education could not explain the relationship between PCS status andAAO.

The mean difference in AAO in bvFTD in accordance with right AC sulcation was 3.4 years. In the context of bvFTD, a disease with an expected survival time from diagnosis of 8–10 years (Garcin et al. 2009), the magnitude of this difference is clinically meaningful. As such, assuming confirmation of our finding, it is recommended that AC sulcation is acknowledged in designing future trials of disease-modifying treatment in bvFTD.

The present study consciously excluded genetic cases of bvFTD as we expected that a possible effect of AC sulcation on bvFTD disease expression may have been compromised by the influence of a strong etiological factor like a dominant mutation with high penetrance. However, since disease-modifying treatments in bvFTD will likely be trailed first in cohorts of mutation carriers, such as in the Genetic Frontotemporal Initiative study, the influence of cingulate morphology on age at symptom onset in genetic cases of bvFTD may warrant attention. Two previous studies (Huster et al. 2007; Wei et al. 2017) identified an interaction between sex and handedness, with right-handed males and left-handed females exhibiting a greater prevalence of leftward PCS asymmetry than left-handed males and right-handed females (Huster et al. 2007; Wei et al. 2017). Although sex was controlled in our study, handedness data were not collected and is a limitation. A further limitation is the lack of neuropathological diagnostic verification. As differing cerebral atrophy patterns are associated with specific neurodegenerative diseases loss of blinding is a potential issue. Our main finding was, however, discovered on an a priori pre-registered intragroup analysis of the bvFTD group, which this factor will not have affected. Additionally, lifetime exposures exist with a potential impact on disease onset in bvFTD; these include but are not limited to occupation, physical exercise, leisure activities, social engagement, and dietary habits. These exposures were not analyzed in this study but have been considered to comprehensively impact upon cognitive reserve (Perneczky et al. 2019; Stern et al. 2020); the potential confounding impact of these exposures is therefore a potential study limitation.

A confirmatory study in a significantly powered novel cohort is required to confirm right ACC sulcation as a disease-modifying factor in sporadic bvFTD. AAO was chosen in this study as a measure of disease expression since it is deemed to be an important feature of the disease (for prognostication and treatment trial design, among others), is robust and data were available for all individuals from all cohorts involved in this study. Future study should explore the relationship with more specific measures of disease expression such as behavioral or neuropsychological profile. Structural and functional studies of the cingulate and paracingulate with respect to sulcation pattern are indicated in order to further understanding of the role of the ACC in sporadic bvFTD and elucidate a potential target for disease-modifying treatment.

In summary, findings presented in the present study suggest that right ACC sulcation may impact upon disease expression in sporadic cases of bvFTD, such that increased right ACC sulcation may delay disease onset.

## Supplementary Material

harper_et_al_supplementary_211111_bhab457

## Notes

We express our gratitude toward all participating subjects, their next of kin, all personnel from the four studies contributing with material to this work, and the funding bodies. *Conflict of Interest*: O.H. has acquired research support (for the institution) from AVID Radiopharmaceuticals, Biogen, Eli Lilly, Eisai, GE Healthcare, Pfizer, and Roche. In the past 2 years, he has received consultancy/speaker fees from AC Immune, Alzpath, Biogen, Cerveau and Roche. E.S. reports no competing interests. J.D.R. has been on a Medical Advisory Board for Alector, Wave Life Sciences, and Prevail Therapeutics as well as performing consultancy for Biogen, Arkuda Therapeutics, Astex, UCB, and Takeda.
