# Correction to: Prenatal gyrification pattern affects age at onset in frontotemporal dementia

**DOI:** 10.1093/cercor/bhad188

**Published:** 2023-05-13

This is a correction to: Luke Harper, Olof Lindberg, Martina Bocchetta, Emily G Todd, Olof Strandberg, Danielle van Westen, Erik Stomrud, Maria Landqvist WaldÖ, Lars-Olof Wahlund, Oskar Hansson, Jonathan D Rohrer, Alexander Santillo, Prenatal gyrification pattern affects age at onset in frontotemporal dementia, *Cerebral Cortex*, Volume 32, Issue 18, 15 September 2022, Pages 3937–3944, https://doi.org/10.1093/cercor/bhab457.

In the originally published version of this manuscript, in the Methods and Materials section, under subheading Participants, there was an error in the following text:

`The bvFTD group consisted of 105 participants (2 possible, 92 probable, and 10 definite bvFTD), 70 males and 35 females with a mean age at scan (AAS) of 66.9 years (SD 8.15) and AAO of 62.2 (SD 8.23). The second group consisted of 110 cognitively healthy controls (HCs), 72 males and 38 females with a Mini Mental State Examination (MMSE) score of ≥26 and mean AAS 62.4 (SD 12.11).

This text has now been corrected, in the online version of the manuscript, to read as follows:

`The bvFTD group consisted of 105 participants (2 possible, 93 probable, and 10 definite bvFTD), 70 males and 35 females with a mean age at scan (AAS) of 66.9 years (SD 8.15) and AAO of 62.2 (SD 8.23). The second group consisted of 110 cognitively healthy controls (HCs), 72 males and 38 females with a Mini Mental State Examination (MMSE) score of ≥26 and mean AAS 62.4 (SD 12.11).'

In addition, the title of the supplementary data file accompanying the manuscript was originally incorrectly given as `Anterior Cingulate Sulcation affects disease expression in Frontotemporal Dementia'. This has now been corrected to `Prenatal gyrification pattern affects age at onset in frontotemporal dementia: Supplementary material'.

