# Supplementary material for: Prenatal gyrification pattern affects age at onset in frontotemporal dementia
Source: Cereb Cortex. 2022 Jan 17;32(18):3937–44. doi: 10.1093/cercor/bhab457 (PMC9476616; doi:10.1093/cercor/bhab457)
Supplement: harper_et_al_supplementary_211111_bhab457 [file harper_et_al_supplementary_211111_bhab457.docx]

**Prenatal gyrification pattern affects age at onset in frontotemporal dementia: Supplementary material**

**Supplementary Material**

**Authors**

Luke Harper^1^, Olof Lindberg^2^, Martina Bocchetta^3^, Emily G Todd^3^, Olof Strandberg^1^, Danielle van Westen^1^, Erik Stomrud^1,4^, Maria Landqvist Waldö^1^ Oskar Hansson^1,4^, Jonathan D Rohrer^3^, Alexander Santillo^1^

^1^Department of Clinical Sciences, Clinical Memory Research Unit, Faculty of Medicine, Lund University, Lund/Malmö, Sweden

^2^Division of Clinical Geriatrics, Karolinska Institute, Stockholm, Sweden

^3^Dementia Research Centre, Department of Neurodegenerative Disease, UCL Queen Square Institute of Neurology, University College London, London, United Kingdom

^4^ Memory Clinic, Skåne University Hospital, Sweden

**Materials and Methods**

**Participants**

The study population was drawn from the following cohorts recruited from the following memory clinics at University Hospitals in Sweden and the United Kingdom: LUPROFS (Lund Prospective Frontotemporal Dementia study), BioFINDER-2 (Skåne University Hospital, Sweden [NCT03174938]), the Longitudinal Investigation of FTD study (University College London, UK) and Empathy in FTD (Lund-Stockholm-Umeå, Sweden).

**Table 1. Study Population Distribution by Cohort**

| **Cohort**  LUPROFS  Empathy in FTD  BioFINDER-2  LIFTD at UCL | **bvFTD**  25  12  3  65 | **AD**  0  1  91  0 | **HC**  13  6  13  78 |
| --- | --- | --- | --- |

**LUPROFS**

Lund Prospective Frontotemporal Dementia Study (LUPROFS) is a longitudinal study of the FTD spectrum disorders conducted at the Memory Clinic at Skåne University Hospital in Lund, Sweden. Patients are diagnosed by a multidisciplinary team after clinical examination, standardized symptom assessment, neuropsychological and neurological examination, blood, CSF and brain MRI analysis. Participants were included between 2009 to 2014. The exclusion criteria included: > 3 lacunar strokes or any number of other type of strokes visible on MRI examination, alcohol addiction, or any other significant neurological or psychiatric comorbidity. CSF biomarker analysis strongly suggestive of Alzheimer´s disease was an additional exclusion criterion for the present study. bvFTD diagnosis was according to the International Behavioral Variant FTD Consortium Criteria (Rascovsky and others 2011). Healthy controls (HC) underwent the same procedures as patients and were required to be asymptomatic regarding cognitive and psychiatric symptoms and normal cognitive performance. The same exclusion criteria as for patients were applied. Ethical approval was given by the Regional Ethical Committee in Lund, Sweden.

**LIFTD**

The Longitudinal Investigation of FTD study (University College London, UK) is a longitudinal study comprised of mixed retrospective and prospective data. Participants were included between 2008 and 2019.

All participants were seen at the National Hospital for Neurology and Neurosurgery specialist cognitive disorders clinic in London, United Kingdom. Study participants underwent a standardised multidiscipline clinical, cognitive and physical assessment as well as MRI imaging. Participants were diagnosed by multidisciplinary team consensus according to International Behavioral Variant FTD Consortium Criteria (Rascovsky and others 2011). Individuals with neurological or psychiatric comorbidities were excluded from study. Ethical approval was given by the London Queen Square NRES Committee.

**BioFINDER-2**

The Swedish BioFINDER-2 study (Palmqvist and others 2020) (clinical trial no. NCT03174938, http/://www.biofinder.se) is a prospective and longitudinal at Lund University, Sweden. All individuals were recruited at a memory clinic and diagnosed by multidisciplinary assessment after clinical and neuropsychological examination, brain MRI, and lumbar puncture with analysis of CSF AD biomarkers. bvFTD diagnosis was made according to the International Behavioral Variant FTD Consortium Criteria (Rascovsky and others 2011). AD diagnosis was made as follows: fulfillment of DSM-5 criteria for dementia (major neurocognitive disorder) due to Alzheimer’s disease (American Psychiatric Association, 2013), a Mini-Mental State Examination (MMSE) score of ≥12 points, fluency in Swedish, and a positive Aβ status as determined using CSF Aβ42/Aβ40 ratio with a cutoff of <0.089, as defined in clinical practice at the Sahlgrenska University Hospital, Mölndal, Sweden. As for Healthy controls, criteria are absence of cognitive symptoms as assessed by a memory clinic physician, Mini-Mental State Examination (MMSE) score of 26-30 at baseline, not fulfill the criteria for MCI or any dementia according to DSM-5 (American Psychiatric Association 2013) and fluency in Swedish.

Subjects were included between 2014 and 2020. Ethical approval was given by the Regional Ethical Committee in Lund, Sweden.

## **Empathy in FTD**

Empathy in FTD is a cross sectional study of social cognition in FTD conducted at Memory Clinics at Skåne University Hospital in Lund, Huddinge Hospital Stockholm, and the neurology clinic of Umeå University Hospital in Sweden. Patients are diagnosed by a multidisciplinary team after clinical examination, standardized symptom assessment, social cognition assessment, neuropsychological examination, blood, CSF and brain MRI analysis. Participants were included between 2016 and 2020. The exclusion criteria were: > 3 lacunar strokes or any number of other type of strokes visible on MRI examination, CSF protein analysis strongly suggestive of Alzheimer´s disease, alcohol addiction, or any other significant neurological or psychiatric comorbidity. BvFTD was diagnosis according to the International Behavioral Variant FTD Consortium Criteria (Rascovsky and others 2011). Healthy controls (HC) underwent the same procedures as patients and were required to be asymptomatic regarding cognitive and psychiatric symptoms with normal cognitive performance. Alzheimer disease patients were diagnosed using the same criteria as in the BioFINDER-2 study (please see above). Ethical approval was given by the Regional Ethical Committee in Stockholm, Sweden.

From the original cohorts, 51 participants were excluded from the bvFTD group after identification of carrier status of either a *C9orf72*, *GRN* or *MAPT* mutation. One healthy control subject was removed from the study due to poor MR image quality. One AD subject was excluded from analysis after identification of a frontal lobe meningioma on MR imaging.

The final study population consisted of 310 subjects across the three sex-matched groups (Chi-squared = 0.09, df = 2, p-value = 0.96) All study subjects gave written informed consent prior to inclusion in their original studies in accordance with local ethics committee requirements.

**Age at Onset (AAO)**

Age at onset was identified as the first date at which typical symptoms of bvFTD (Rascovsky and others 2011) became apparent to either the patient or their nearest family member or a concern about a participant’s behaviour change indicating symptomatology of bvFTD was highlighted by a third party e.g., an employer. This information was recorded through interviewing of participants and nearest family members at memory clinics following initial referral.

**Age at Scan (AAS)**

Age at scan was determined by the date of the initial MRI examination performed on a participant in conjunction with referral to a memory clinic or study inclusion.

**MRI acquisition and Software**

High resolution volumetric whole brain T1-weighted MR-images were obtained using 3.0 Tesla Magnetic resonance imaging machines.

LUPROFS imaging was performed using a Philips Achieva 3.0T scanner equipped with an eight-channel head coil. A T1-weighted 3D volumetric sequence was also acquired with a resolution of 1x1x1 mm^3^, TR 8.3 ms, TE 3.84 ms, FOV 256´256´175 mm^3^.

LIFTD images were acquired on 3T Trio MRI scanner (Siemens, Erlangen, Germany, TR = 2200 ms, TE = 2.9 ms, spatial resolution = 1.1 mm, flip-angle = 8°) and on 3T Prisma MRI scanner (Siemens, Erlangen, Germany, TR = 2000 ms, TE = 2.93 ms, acquisition matrix = 256 × 256, spatial resolution = 1.1 mm, flip-angle = 8°).

BioFINDER-2 imaging was performed on a 3.0T Siemens Tim Trio scanner (Siemens Medical Solutions, Erlangen, Germany). Images were acquired using a MPRAGE sequence (in-plane resolution = 1×1 mm^2^, slice thickness = 1.2 mm, TR = 1950 ms, TE = 3.37 ms, flip-angle = 9°).

Empathy in FTD imaging was performed using a 3.0T Siemens Magnetom Prisma Fit scanner. (Siemens Medical Solutions, Erlangen, Germany) using MPRAGE sequence (in-place resolution = 1×1 mm^2^, slice thickness = 1.2 mm, TR = 2300 ms, TE = 2.98 ms, flip-angle = 9°)

Prior to analysis T1 images were anonymised and visually inspected for the presence of significant artefact or pathology which may have obscured paracingulate sulcation visualisation and classification. Images were prepared so that the x axis in the sagittal plane was aligned with the bicommissural line (AC–PC). Additional y and z axis rotational corrections were performed in order to ensure optimal orientation for analysis.

**Preregistration**

The pre-registration protocol was registered with Open Science Framework Registries and may be accessed directly at <https://osf.io/h6t2z>.

**Power Calculation**

Assuming a similar sulcation difference may be found in this study, data from two studies in Schizophrenia (Le Provost and others 2003; Yücel and others 2002) were utilized to perform power calculations using Fisher´s exact test. Two hypotheses were accounted for (H1: bvFTD vs HC, H2: BvFTD vs AD), as such a Bonferroni correction was performed, alpha 0.025 (0.05/2), power 0.8. Calculations identified a desired population of between 47 and 96 subjects per group.

Power calculations were performed in G*Power, Statistical power analysis program (Faul and others 2007) using Fishers exact test.

**Utilising data from Yucel et al(Yücel and others 2002)**

Left Hemisphere PCS frequency

Control 84/100

Schizophrenia 57/101

Power calculation: Alpha 0.025 (H1. BvFTD vs HC. H2. bvFTD vs AD). One-sided hypothesis. Power 0.8. Fishers exact test yields 47 left hemispheres per cohort required for a sufficiently powered study.

**Utilising data from Le Provost et al(Le Provost and others 2003)**

Left Hemisphere PCS frequency

Control 66/100

Schizophrenia 45/100

Power calculation. Alpha 0.025 (H1. BvFTD vs HC. H2. bvFTD vs AD). One-sided hypothesis. Power 0.8. Fishers exact test yields 96 left hemispheres per cohort required for a sufficiently powered study.

**Paracingulate Sulcus Measurement and Classification Criteria**

Garrison’s established protocol for PCS identification and measurement was refined and utilised for PCS analysis in all hemispheres.(Garrison and others 2015)

A binary sulcation classification was utilised where the PCS was categorized as either “present” (≥20 mm) or “absent” (≤19 mm) as is standard amongst PCS classification protocols.(Del Maschio and others 2019; Garrison and others 2015; Le Provost and others 2003; Ono and others 1990; Yücel and others 2002)

The cingulate sulcus (CS) is identified 4 mm laterally from the midline (x = 0). The PCS is identified as the sulcus running predominantly horizontally, dorsal and parallel to the CS. The PCS depth must be clearly visible in four or more consecutive sagittal slices (>4 mm). The anterior limit of the PCS is identified as the point at which the sulcus begins to move posteriorly and parallel to the CS from an imaginary line perpendicular to the AC–PC line (Yücel and others 2001). The PCS is measured from this point using MANGO’s (Multi-image Analysis GUI, v 4.0, <http://ric.uthscsa.edu/mango/mango.html>, The University of TexasHealth Science Center) “Trace Line” function until its end point, the point where the sulcus is interrupted by a distinct predominantly vertical gyri deemed non-PC in nature. The PCS may fall outside of the first quadrant but must originate in the first quadrant on a sagittal plane where x0, y0 marks the point of the anterior commissure after images have been aligned in the AC-PC plane.

The measurement protocol was modified in this study such that discontinuous PCS lacking an individual segment ≥20 mm in length were classified as “absent”. This decision was made as raters in this study found measurement of these structures to be inconsistent and unreliable in pre-study rater training. In addition, present PCS were required to be visible on four consecutive sagittal slices. Measurement was performed on the sagittal slice where the anteroposterior PCS length was shortest of the four longest consecutive sagittal slices where the PCS could be identified. This adaption meant that sulcation length was maintained throughout a depth of four sagittal slices which we believe to have increased the reliability of detection of a true PCS.

**Rating and Reliability Testing**

Sulcation ratings were performed by two raters, LH and AS, who were blinded to individual’s age, gender and study group. Prior to study reliability studies were conducted using the methods described above in an independent cohort of 30 healthy individuals. Intra-rater agreement, LH vs LH was 98.33%, Cohens Kappa 0.96. Inter-rater agreement AS vs LH was 94.44%, Cohens Kappa 0.85.

In study analysis, after independent sulcation rating similar rater disagreement was observed in each of the study groups, (bvFTD; 19, AD; 18, HC; 15). Rater disagreement was resolved by consensus and the primary rater’s (LH) rating was changed in the following cases, (bvFTD; 6, AD; 8, HC; 5).

**References**

American Psychiatric Association. 2013. Diagnostic and Statistical Manual of Mental Disorders. Washington, DC.

Del Maschio N, Sulpizio S, Fedeli D, Ramanujan K, Ding G, Weekes BS, Cachia A, Abutalebi J. 2019. ACC Sulcal Patterns and Their Modulation on Cognitive Control Efficiency Across Lifespan: A Neuroanatomical Study on Bilinguals and Monolinguals. Cereb Cortex 29(7):3091-3101.

Faul F, Erdfelder E, Lang AG, Buchner A. 2007. G*Power 3: a flexible statistical power analysis program for the social, behavioral, and biomedical sciences. Behav Res Methods 39(2):175-91.

Garrison JR, Fernyhough C, McCarthy-Jones S, Haggard M, Simons JS. 2015. Paracingulate sulcus morphology is associated with hallucinations in the human brain. Nat Commun 6:8956.

Le Provost JB, Bartres-Faz D, Paillere-Martinot ML, Artiges E, Pappata S, Recasens C, Perez-Gomez M, Bernardo M, Baeza I, Bayle F et al. . 2003. Paracingulate sulcus morphology in men with early-onset schizophrenia. Br J Psychiatry 182:228-32.

Ono M, Kubik S, Abernathey CD. 1990. Atlas of the cerebral sulci.

Palmqvist S, Janelidze S, Quiroz YT, Zetterberg H, Lopera F, Stomrud E, Su Y, Chen Y, Serrano GE, Leuzy A et al. . 2020. Discriminative Accuracy of Plasma Phospho-tau217 for Alzheimer Disease vs Other Neurodegenerative Disorders. Jama 324(8):772-781.

Rascovsky K, Hodges JR, Knopman D, Mendez MF, Kramer JH, Neuhaus J, van Swieten JC, Seelaar H, Dopper EG, Onyike CU et al. . 2011. Sensitivity of revised diagnostic criteria for the behavioural variant of frontotemporal dementia. Brain 134(Pt 9):2456-77.

Yücel M, Stuart GW, Maruff P, Velakoulis D, Crowe SF, Savage G, Pantelis C. 2001. Hemispheric and gender-related differences in the gross morphology of the anterior cingulate/paracingulate cortex in normal volunteers: an MRI morphometric study. Cereb Cortex 11(1):17-25.

Yücel M, Stuart GW, Maruff P, Wood SJ, Savage GR, Smith DJ, Crowe SF, Copolov DL, Velakoulis D, Pantelis C. 2002. Paracingulate morphologic differences in males with established schizophrenia: a magnetic resonance imaging morphometric study. Biol Psychiatry 52(1):15-23.
